# The SIRT7-nucleolus connection in cancer: ARF enters the fray

**DOI:** 10.1080/23723556.2024.2381287

**Published:** 2024-07-17

**Authors:** Shahriar Tarighi, Poonam Kumari, Alejandro Vaquero, Thomas Braun, Alessandro Ianni

**Affiliations:** a Department of Cardiac Development and Remodeling, Max-Planck-Institute for Heart and Lung Research, Bad Nauheim, Germany; b Chromatin Biology Laboratory, Josep Carreras Leukaemia Research Institute (IJC), Barcelona, Spain; c Member of The German Center for Lung Research (DZL), Member of The Cardio-Pulmonary Institute (CPI), Giessen, Germany

**Keywords:** Sirtuin 7, SIRT7, nucleolus, cancer

## Abstract

The nucleolar enzyme sirtuin 7 (SIRT7) promotes cancer progression in certain malignancies, likely in part by controlling ribosome biosynthesis. Recently, we discovered that SIRT7 destabilizes the cyclin dependent kinase inhibitor 2A (CDKN2A, known as ARF) within the nucleolus, aiding cancer progression. We propose that targeting nucleolar SIRT7 offers promise for new anti-cancer therapies.

## Author´s view

Sirtuin 7 (SIRT7), a member of the mammalian family of NAD^+^-dependent histone/protein deacetylases, collectively known as sirtuins, regulates various biological functions and has recently been recognized as a critical factor in controlling cancer progression.^1^

Besides specific tumors where SIRT7 appears to act as a tumor suppressor, in the vast majority of cases- including liver, pancreas, intestine, colorectal, skin, lung, and thyroid cancers- SIRT7 functions as a prominent pro-tumorigenic factor. This is achieved by controlling various molecular pathways involved in cancer cell proliferation, migration, and survival. Consistently, these tumors often exhibit elevated SIRT7 levels compared to healthy tissues, and higher SIRT7 expression correlates with more severe phenotypes and poorer prognosis.^2^

SIRT7 accumulates within the nucleolus, a nuclear compartment primarily responsible for ribosome biogenesis. Recent work suggests that SIRT7 exerts pro-tumorigenic functions, at least in part, by regulating nucleolar activities.^1,2^

Ribosomes are complex macromolecules responsible for mRNA translation and protein synthesis and are constituted of ribosomal RNA (rRNA) along with ribosomal proteins (RPs). Highly proliferating cancer cells demand increased protein synthesis to sustain growth, necessitating enhanced ribosome biogenesis. Accordingly, numerous oncogenes promote cancer growth by stimulating ribosome biogenesis, whereas tumor suppressors typically inhibit this process.^3^

**Figure 1.**
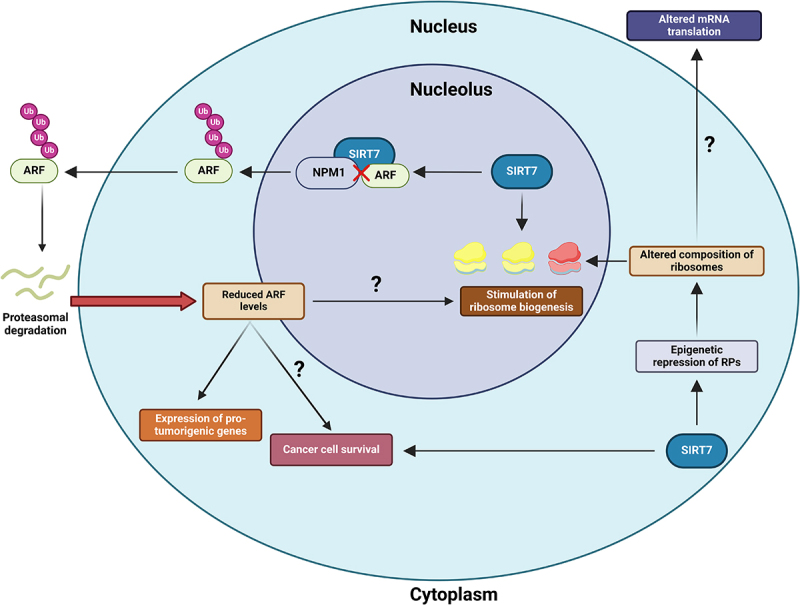
Scheme depicting the potential nucleolar mechanisms employed by sirtuin 7 (SIRT7) to sustain cancer progression. SIRT7 stimulates ribosome biogenesis by activating various mechanisms involved in this process, potentially promoting cancer cell growth. Additionally, SIRT7 epigenetically represses the expression of specific ribosomal proteins (RPs), which could alter ribosome composition to influence the translation of specific mRNAs involved in tumorigenesis. Within the nucleolus, SIRT7 destabilizes the cyclin dependent kinase inhibitor 2A (CDKN2A), specifically the splicing variant known as Alternative Reading Frame (ARF). SIRT7 directly interacts with ARF, interrupting its interaction with nucleophosmin 1 (NPM1). This event facilitates the exclusion of ARF from the nucleolus, promoting ARF ubiquitination and proteasomal-dependent degradation. SIRT7-mediated inhibition of ARF promotes the expression of pro-tumorigenic genes and may attenuate ARF’s roles in suppressing cancer cell death and ribosome biogenesis, thereby fueling cancer progression. Question marks denote hypothesized functions that require experimental validation.

SIRT7 appears to promote cancer growth, at least partially, by facilitating ribosome biogenesis through stimulating various steps of the process, such as rRNA transcription and maturation.^1,2^ Conversely, SIRT7 also epigenetically represses the expression of specific RPs, potentially altering ribosome stoichiometry. Altered ribosome composition can influence the affinity of ribosomes for specific mRNAs encoding cancer-related proteins, such as tumor suppressors, ultimately altering their translation and promoting cancer progression. Thus, it was proposed that SIRT7 contributes to tumor progression through this mechanism (Figure 1).^2,4^

Besides its role in ribosome biogenesis, the nucleolus has recently been recognized as a key player in other cellular functions. Only around 30% of nucleolar proteins are involved in ribosome biogenesis, while the rest participate in processes such as apoptosis, cell cycle regulation, and DNA repair. Confinement within the nucleolus significantly influences the turnover and activity of these molecules.^5,6^ The cyclin dependent kinase inhibitor 2A, specifically the splicing variant commonly known as Alternative Reading Frame (ARF; p14ARF in humans and p19ARF in mice), serves as a well-known example of this regulatory mechanism.

ARF is a potent nucleolar tumor suppressor that inhibits cancer progression by orchestrating a wide range of downstream signaling cascades. Depletion of ARF in mice dramatically accelerates tumorigenesis. Moreover, genomic alterations or epigenetic silencing of the *CDKN2A* locus encoding ARF, as well as the activation of mechanisms promoting ARF protein degradation, have been reported in various human malignancies.^7,8^

Interaction of ARF with nucleolar proteins such as nucleophosmin 1 (NPM1) facilitates ARF accumulation in the nucleolus. This process enhances ARF’s stability by preventing interaction with specific ubiquitin ligases responsible for its ubiquitination and subsequent proteasomal degradation outside the nucleolus. Intriguingly, various oncogenes and carcinogens have been found to destabilize ARF by disrupting its binding with NPM1.^7,8^

Moreover, common mutations in the *NPM1* gene, which frequently occur in acute myeloid leukemia (AML), result in a mutated form of NPM1 that is largely absent from the nucleolus. This leads to decreased ARF accumulation in this compartment, rendering ARF more vulnerable to degradation and thereby facilitating AML progression.^9^

Recently, we demonstrated that SIRT7 directly interacts with ARF in lung cancer cells and promotes its proteasomal-dependent degradation by inhibiting its interaction with NPM1.^7^ This event prevents ARF from repressing genes involved in stimulation of cancer cells growth, thereby facilitating lung cancer progression (Figure 1).^7^

We observed increased SIRT7 levels in human lung tumors compared to healthy controls, an event that correlates with decreased ARF protein. Our bioinformatics analyses of transcriptomic data from human lung cancer samples demonstrated that high SIRT7 expression in tumors correlates with increased expression of genes normally repressed by ARF, exclusively in tumors harboring an intact *CDKN2A* locus.^7^

The destabilization of ARF by SIRT7 may also influence other functions of ARF crucially involved in cancer progression. For instance, ARF is a potent inhibitor of ribosome biogenesis,^10^ suggesting that SIRT7 may promote this process, at least in part, by inhibiting ARF. Additionally, ARF plays a significant role in mediating apoptosis in cancer cells,^8^ while SIRT7 frequently supports cancer cell survival, especially in response to anticancer treatments.^2^ This evidence suggests that SIRT7 might contribute to drug resistance by inhibiting ARF (Figure 1).

Many functions of SIRT7 rely on modulation of downstream signaling cascades *via* deacetylation of specific targets. Consistently, inhibitors of SIRT7 exhibit efficacy in reducing tumor progression in mice.^2^ However, in our study, we demonstrate that SIRT7 does not rely on its deacetylation activity to destabilize ARF but rather competes for NPM1-ARF binding sites to interrupt their interaction.^7^ This evidence suggests that in tumors with intact ARF expression, innovative approaches such as small-molecule-induced protein degradation methods – specifically, proteolysis-targeting chimeras (PROTACs) and hydrophobic tagging technologies – might represent more effective therapeutic strategies for targeting SIRT7. These methods may be particularly advantageous as they lower overall protein levels rather than solely inhibiting catalytic activity.^7^

Similar to ARF, SIRT7 may regulate the activity of other nucleolar proteins to influence tumor progression through mechanisms that may or may not depend on its catalytic activity. This hypothesis gains support from mass spectrometry analyses that have identified numerous SIRT7-interacting partners, many of which accumulate in the nucleolus.^2^

Further comprehensive studies are essential to understand the role of SIRT7-mediated regulation of the nucleolus in cancer progression. Clarifying these functions will be crucial in determining whether targeting SIRT7 localization in this compartment could pave the way for innovative anti-cancer therapies.

## Data Availability

Data sharing not applicable – no new data generated
